# Perioperative Prophylactic Chemotherapy of *Echinococcus Granulosus*: Determination of Minimum Effective Length of Albendazole Therapy in *in Vitro* Protoscolex Culture

**DOI:** 10.1155/1990/67385

**Published:** 1990

**Authors:** D. H. Taylor, D. L. Morris, K. S. Richards

**Affiliations:** 1 Department of Surgery University Hospital Nottingham NG7 2UH United Kingdom; 2 Department of Biological Sciences University of Keele Keele Staffs. ST5 5BG United Kingdom

## Abstract

Protoscoleces of *Echinococcus granulosus* were cultured *in vitro* in 500, 250 or 100 *μ*g/1 albendazole sulphoxide for 1, 3, 7, 10, 14d and then ‘recued’ (R) into drug-free medium for the remainder ofthe culture period. Successful minimum lengths of therapy were much longer than for praziquantel, and only at 500*μ*g/1 was the 10dR treatment as effective as continuous therapy for 28d. Treatment with 100 *μ*g/1 both in continuous culture and in the ‘R’ experiments was ineffective over a 35d period. The results are compared with those from similar experiments using praziquantel.


*HPB Surgery*, 1990, Vol. 2, pp. 159-164
Reprints available directly from the publisher
Photocopying permitted by license only
© 1990 Harwood Academic Publishers GmbH
Printed in the United Kingdom

### PERIOPERATIVE PROPHYLACTIC CHEMOTHERAPY OF *ECHINOCOCCUS GRANULOSUS*: DETERMINATION OF MINIMUM EFFECTIVE LENGTH OF ALBENDAZOLE THERAPY IN *IN VITRO* PROTOSCOLEX CULTURE

D.H. TAYLOR[1], D.L. MORRIS[1] and K.S. RICHARDS[2]

[1]*Department of Surgery, University Hospital, Nottingham NG7 2UH, UK.*
[2]*Department of Biological Sciences, University of Keele, Keele, Staffs. ST5 5BG, UK.*

*(Received 15 July 1989)*

Protoscoleces of *Echinococcus granulosus* were cultured *in vitro* in 500, 250 or 100 μg/l albendazole sulphoxide for 1, 3, 7, 10, 14d and then 'rescued' (R) into drug-free medium for the remainder of the culture period. Successful minimum lengths of therapy were much longer than for praziquantel, and only at 500 μg/l was the 10dR treatment as effective as continuous therapy for 28d. Treatment with 100 μg/l both in continuous culture and in the 'R' experiments was ineffective over a 35d period. The results are compared with those from similar experiments using praziquantel.

KEY WORDS: Albendazole, praziquantel, protoscoleces

### INTRODUCTION

Chemotherapy against hydatid disease in man is not without risk, and it is clearly important to know the minimum length of time required for effective therapy. This paper studies the duration of exposure to albendazole needed to kill protoscoleces, as would be necessary in a prophylactic course in patients following operation/spillage. We have previously reported that albendazole sulphoxide, the active metabolite of albendazole, is an effective *in vitro* protoscolicidal agent. Approximately 250 μg/l albendazole sulphoxide is required to produce a significant reduction in viability after 31 days[1,2]. We have also found praziquantel to be a most active protoscolicidal agent and have demonstrated that in *in vitro* the effect of comparatively short-term exposure to the drug causes irreversible damage, and that protoscoleces so treated and then 'rescued' into drug-free media continue to die[3]. It was therefore decided to study short lengths of exposure to various concentrations of albendazole sulphoxide followed by the 'rescue' of the protoscoleces into drug-free medium to establish the minimum exposure time necessary for effective treatment by albendazole sulphoxide.

### MATERIALS AND METHODS

Protoscoleces were collected from both hepatic and pulmonary hydatid cysts in naturally infected sheep obtained from a local abattoir and maintained at 37°C in NCTC135 (Gibco) with 20% heat-inactivated foetal calf serum (Gibco) as previously described[4]. Only cultures with an initial viability of over 95% as judged by microscopy/eosin exclusion were used. Albendazole sulphoxide (Smith, Kline & French) concentrations of 500, 250 and 100 μg/l were studied together with controls to which an identical volume of solvent (50% methanol) had been added. The final concentration of methanol in the culture medium of both test and control tubes was 2.5 ml/l and this has no significant effect on protoscolex viability[5]. A minimum of 10 replicates at each drug concentration was performed. All media were changed and protoscolex viability assessed by microscopy/eosin exclusion every 3–4 days.

At days 1, 3, 7, 10 and 14 individual tubes of treated protoscoleces at each concentration were 'rescued' into drug-free medium for the remainder of the experimental period (28 days for 500 μg/l, 35 days for 250 and 100 μg/l). These will be referred to as 1dR (1 day Rescue), 3dR, 7dR etc. respectively in contrast to tubes receiving continuous drug therapy.

Statistical analysis of viability data for control and treated cultures was done using a GLIM statistical package (GLIM 3.77 update), 1985, Royal Statistical Society, London) to fit logistic regression lines with the proportion of alive protoscoleces as the dependent variable and the number of days in *in vitro* culture as the independent variable, with treatments groups as a factor to allow cross treatment comparisons.

### RESULTS

Figures 1–3 present the viability of protoscoleces treated with 500, 250 and 100 μg/l albendazole sulphoxide and rescued into drug-free medium at days 1, 3, 7, 10 and 14 together with control and continuous therapy cultures.

At a concentration of 500 μg/l albendazole sulphoxide, neither 1 nor 3 days culture with the drug (1dR and 3dR) before rescue caused any significant reduction in protoscolex viability over the 28 day culture period. The 7dR results showed a strong trend towards being different from the controls but were not significantly different ($p < 0.1$). The 10dR treatment not only significantly reduced protoscolex viability when compared with the controls ($p < 0.01$) but was also significantly better than the 7dR therapy ($p < 0.05$). Likewise, 14dR was significantly more effective than both controls and 10dR ($p < 0.01$). Continuous therapy was more effective than controls, 1dR, 3dR and 7dR ($p < 0.01$), but not significantly more than 10dR and 14dR ($p < 0.1$), though a strong trend was apparent.

At a concentration of 250 μg/l of albendazole sulphoxide, only 1dR was not significantly different from the control (3dR $p > 0.025$; 7dR, 10dR and 14dR $p < 0.001$), and each increase in duration of drug treatment achieved a significantly greater reduction in protoscolex viability.

The apparently greater effect of albendazole 250 μg/l compared with 500 μg/l was due to a larger SD in the 500 μg/l controls and if the horizontal 'end viability' portion of the percentage viability curves are studied it will be seen that whilst Alb Sx 500 μg/l achieved viability of 20% or less after 7, 10, 14 or continuous Alb Sx μg/l 7dR achieved approximately 40%, 10dR approximately 30% and it was only 14dR and continuous therapy that achieved 20% or less.

At a concentration of 100 μg/l of albendazole sulphoxide there was no significant difference between any of the experimental groups and the control cultures, even at the end of the 35 day culture period.

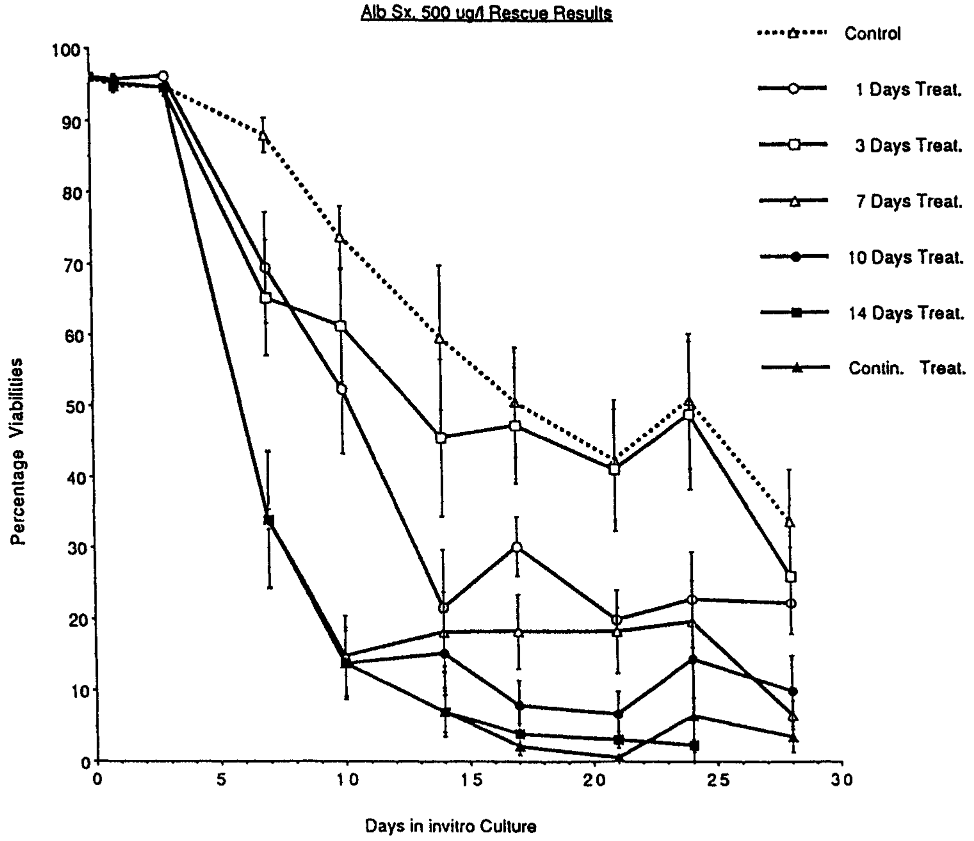

**Figure 1.** Effect of 500 μg/l albendazole sulphoxide for 28d, and for 1, 3, 7, 10, 14d followed by drug-free culture on the viability of protoscoleces of *E. granulosus*.

### DISCUSSION

It has already been established that albendazole sulphoxide is protoscolicidal when used *in vitro* over a 30 day period[1]. It was not clear, however, whether this length of treatment was necessary for a drug-induced injury to cause death of the protoscoleces or whether continuous treatment was actually necessary to achieve the lethal effect.

*In vitro* culture of protoscoleces of *E. granulosus* has allowed us to study various drug concentrations and lengths of therapy. It must be accepted however that each experiment is different and that because of batch to batch variations in both the viability and survival of protoscoleces, controls must be maintained in each experiment and reduction in viability of treated cultures compared with the viability of control cultures within the same experiment.

The present study has shown that continued exposure to albendazole sulphoxide is required, and that after drug withdrawal there is little if any further decrease in

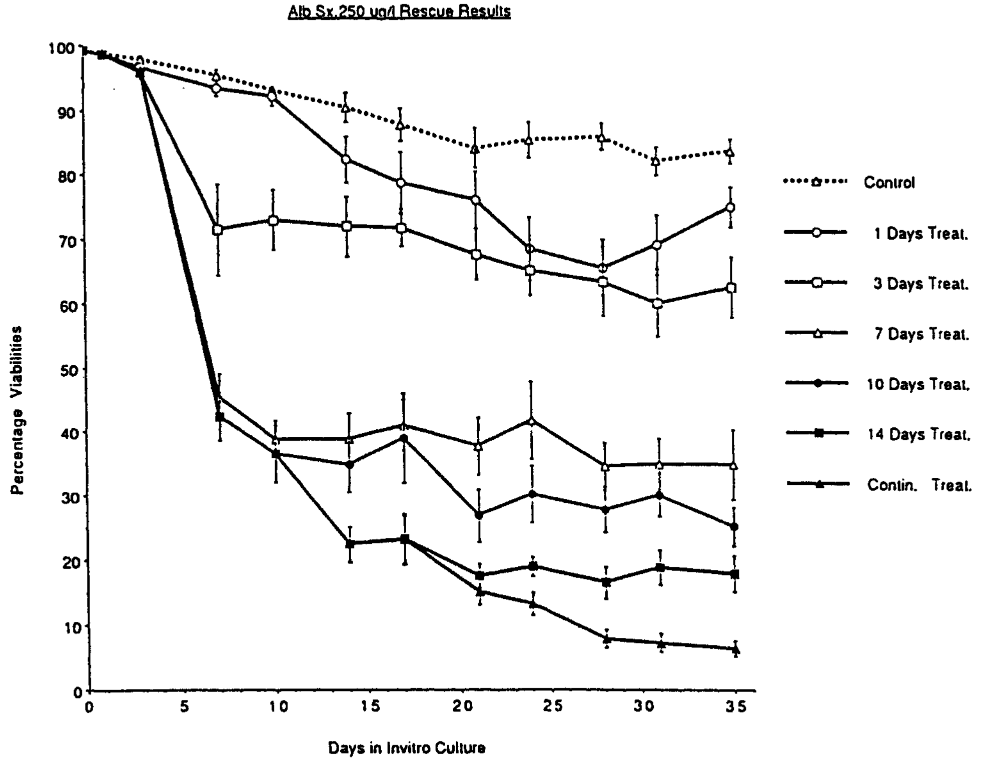

**Figure 2.** Effect of 250 $\mu$g/l albendazole sulphoxide for 35d, and for 1, 3, 7, 10, 14d followed by drug-free culture on the viability of protoscoleces of *E. granulosus*.

protoscolex viability. This is different from praziquantel where very short lengths of treatment are lethal[3] and exposure times of as short as 10 min are effective at very high concentrations[6]. This difference between albendazole, a benzimidazole carbamate, and praziquantel, an isoquinoline, is probably related to the different mechanism of action of these compounds. Praziquantel is known to have a disruptive effect on the tegument of both the adult and protoscolex stage of *E. granulosus*[7–10] whereas benzimidazoles are thought to bind to tubulin[11] and inhibit its polymerization, and thus have a more gradual effect on parasite tissues. The results of the current 'rescue' type experiments would seem to support this in that prolonged therapy with albedazole sulphoxide seems to be necessary in order to achieve the same degree of protoscolicidal efficacy as that brought about by much shorter periods of treatment with praziquantel. The administration of albendazole as an intraoperative agent is therefore unlikely to be effective, but its possible role in postoperative therapy might still be considered.

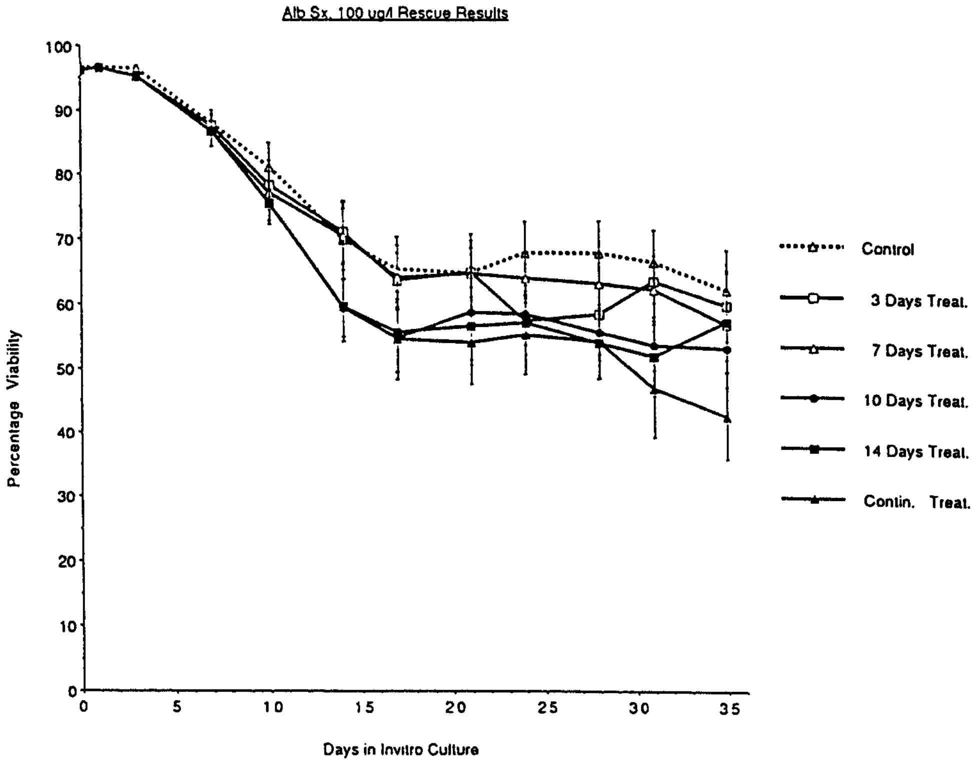

**Figure 3.** Effect of 100 μg/l albendazole sulphoxide for 35d, and for 1, 3, 7, 10, 14d followed by drug-free culture on the viability of protoscoleces of *E. granulosus.*

### *Acknowledgements*

Smith, Kline & French are thanked for their financial support and for the supply of albendazole sulphoxide.

### *References*

1 Chinnery, J.B. and Morris, D.L., Effect of albendazole sulphoxide on viability of hydatid protoscoleces *in vitro, Trans. Roy. Soc. Trop. Med. Hyg.*, 1986; **80**: 815–817.

2 Morris, D.L., Chinnery, J.B. and Ubhi, C., A comparison of the effects of albendazole, its sulphone metabolite and mebendazole on viability of *Echinococcus granulosus* protoscoleces in an *in vitro* culture system, *Trans. Roy. Soc. Trop. Med. Hyg.,*, 1987; **81**: 804–806.

3 Morris, D.L., Taylor, D.H., Daniels, D., *et al.*, Determination of the minimum time of praziquantel therapy required for the *in vitro* treatment of protoscoleces of *Echinococcus granulosus, J. Helminth.*, 1988; **62**: 10–14.

4 Taylor, D.H., Morris, D.L. and Richards, K.S., Combination chemotherapy of *Echinococcus granulosus - in vitro* studies, *Trans. Roy. Soc. Trop. Med. Hyg.*, 1988 **82**: 263–264.

5 Taylor, D.H. and Morris, D.L., *In vitro* culture of *Echinococcus multilocularis*: protoscolicidal action of praziquantel and albendazole sulphoxide, *Trans. Roy. Soc. Trop. Med. Hyg.*, 1988; **82**: 265–267.
6 Richards, K.S., Riley, E.M., Taylor, D.H. and Morris, D.L., Studies on the effect of short term, high dose praziquantel treatment against protoscoleces of ovine and equine *Echinococcus granulosus* within the cyst, and *in vitro, Trop. Med. Parasit.*, 1988; **39**: 269–272.
7 Becker, B., Mehlhorn, H., Andrews, P. and Thomas, H., Ultrastructural investigations on the effect of praziquantel on the tegument of five species of cestodes, *Z. Parasitenkd*, 1981; **64**: 257–269.
8 Conder, G.A., Marchiondo, A.A. and Andersen, F.L., Effect of praziquantel on adult *Echinococcus granulosus in vitro*: scanning electron microscopy, *Z. Parasitenkd*, 1981; **66**: 191–199.
9 Morris, D.L., Richards, K.S. and Chinnery, J.B., Protoscolicidal effect of praziquantel - *in vitro* and electron microscope studies on *Echinococcus granulosus, J. Antimicr. Chem.*, 1986; **18**: 687–691.
10 Morris, D.L., Taylor, D.H., Daniels, D. and Richards, K.S., Determination of minimum effective concentrations of praziquantel in *in vitro* cultures of protoscoleces of *Echinococcus granulosus, Trans. Roy. Soc. Trop. Med. Hyg.*,., 1987; **81**: 494–497.
11 Laclette, J.P., Guerra, G. and Zetina, C., Inhibition of tubulin polymerization by mebendazole, *Biochem. Biophys. Res. Comm.*, 1980; **92**: 417–423.

*(Accepted by S. Bengmark 27 July 1989)*

